# Supplementary figures and images for: Chemical nucleases are a robust alternative for RNase H cleavage of human ribosomal RNA
Source: PLoS One. 2025 Feb 24;20(2):e0318697. doi: 10.1371/journal.pone.0318697 (PMC11849838; doi:10.1371/journal.pone.0318697)

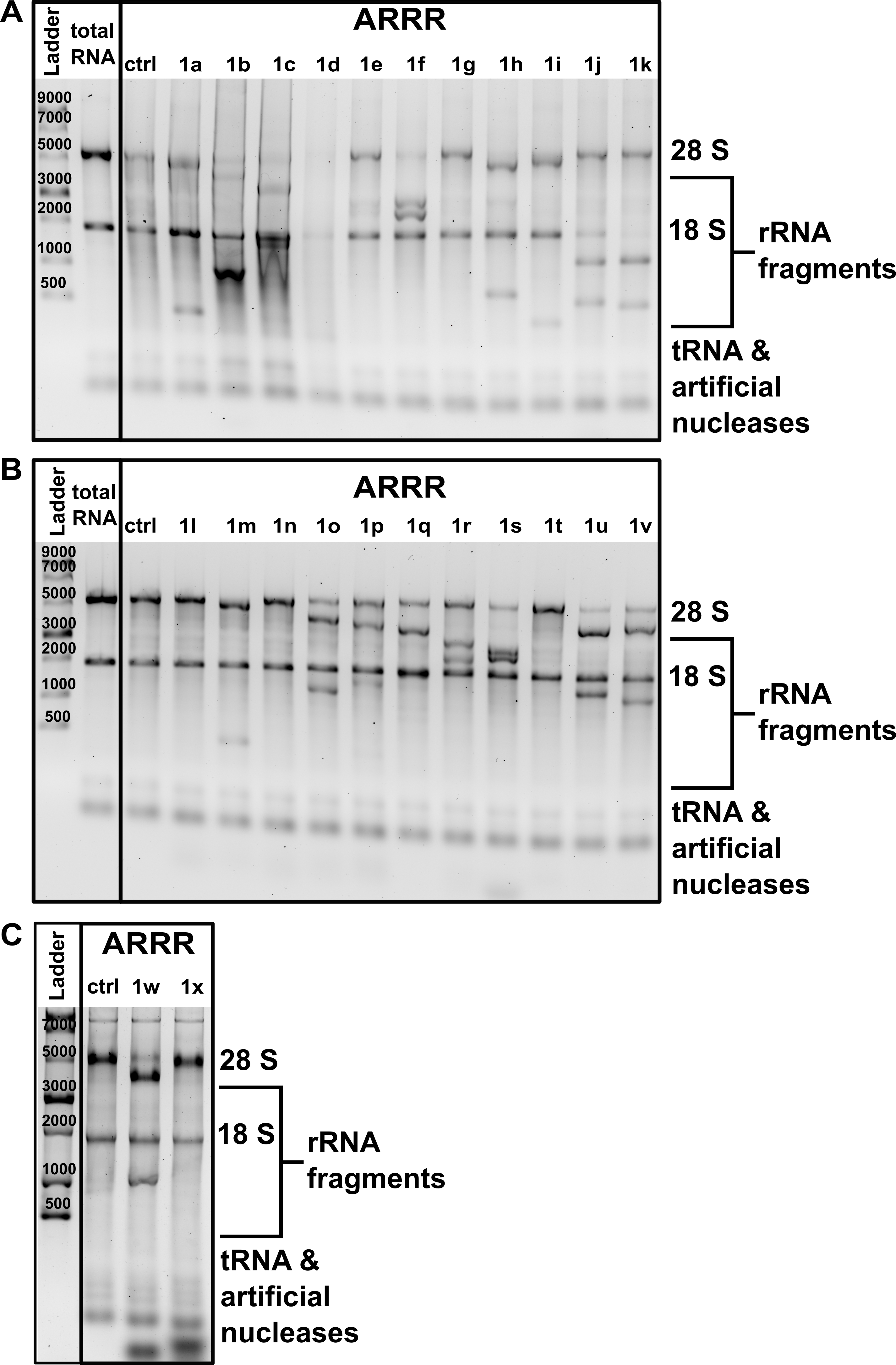

Supplement: S1 Fig — Total RNA and the 5’-conjugated probes 1–24 corresponding cleavage product were monitored on a 2% Agarose Gel. Stained with GelRed. (TIF) [file pone.0318697.s001.tif]

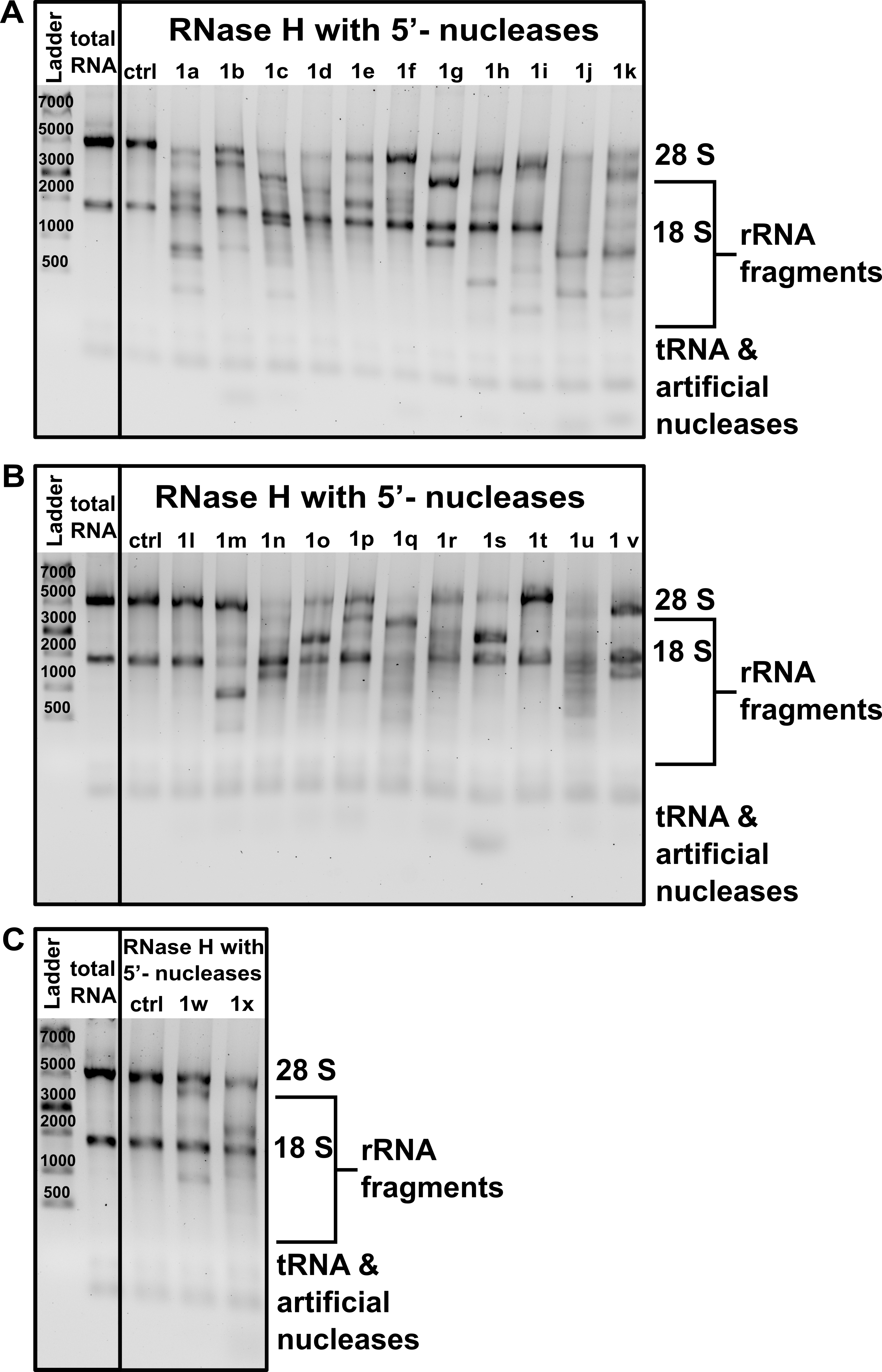

Supplement: S2 Fig — Total RNA and the 5’-conjugated probes 1 – 24 corresponding digestion product were monitored on a 2% Agarose Gel. Stained with GelRed. (TIF) [file pone.0318697.s002.tif]

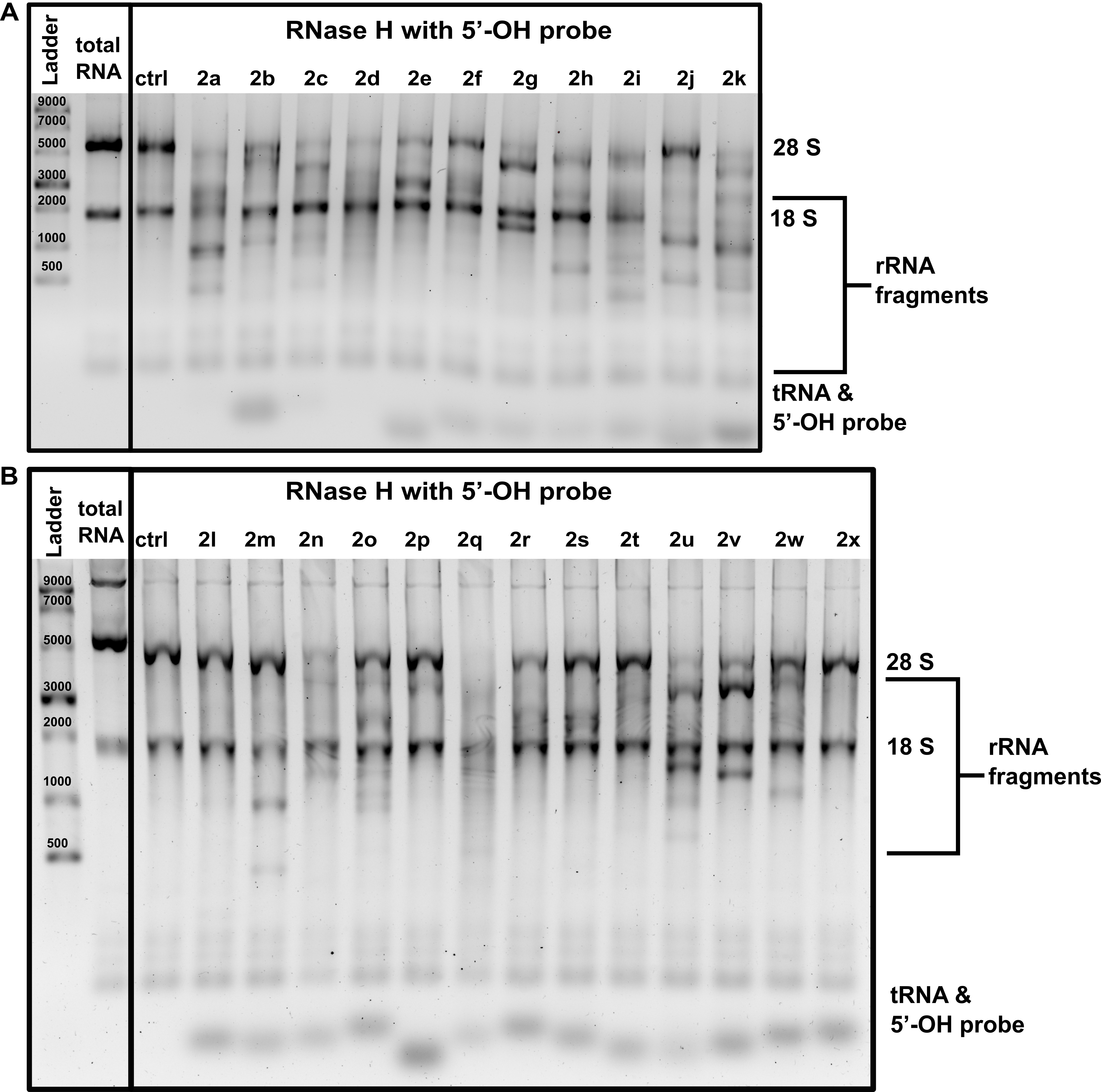

Supplement: S3 Fig — Total RNA and the 5’-OH probes 1 – 24 corresponding digestion product were monitored on a 2% Agarose Gel. Stained with GelRed. (TIF) [file pone.0318697.s003.tif]

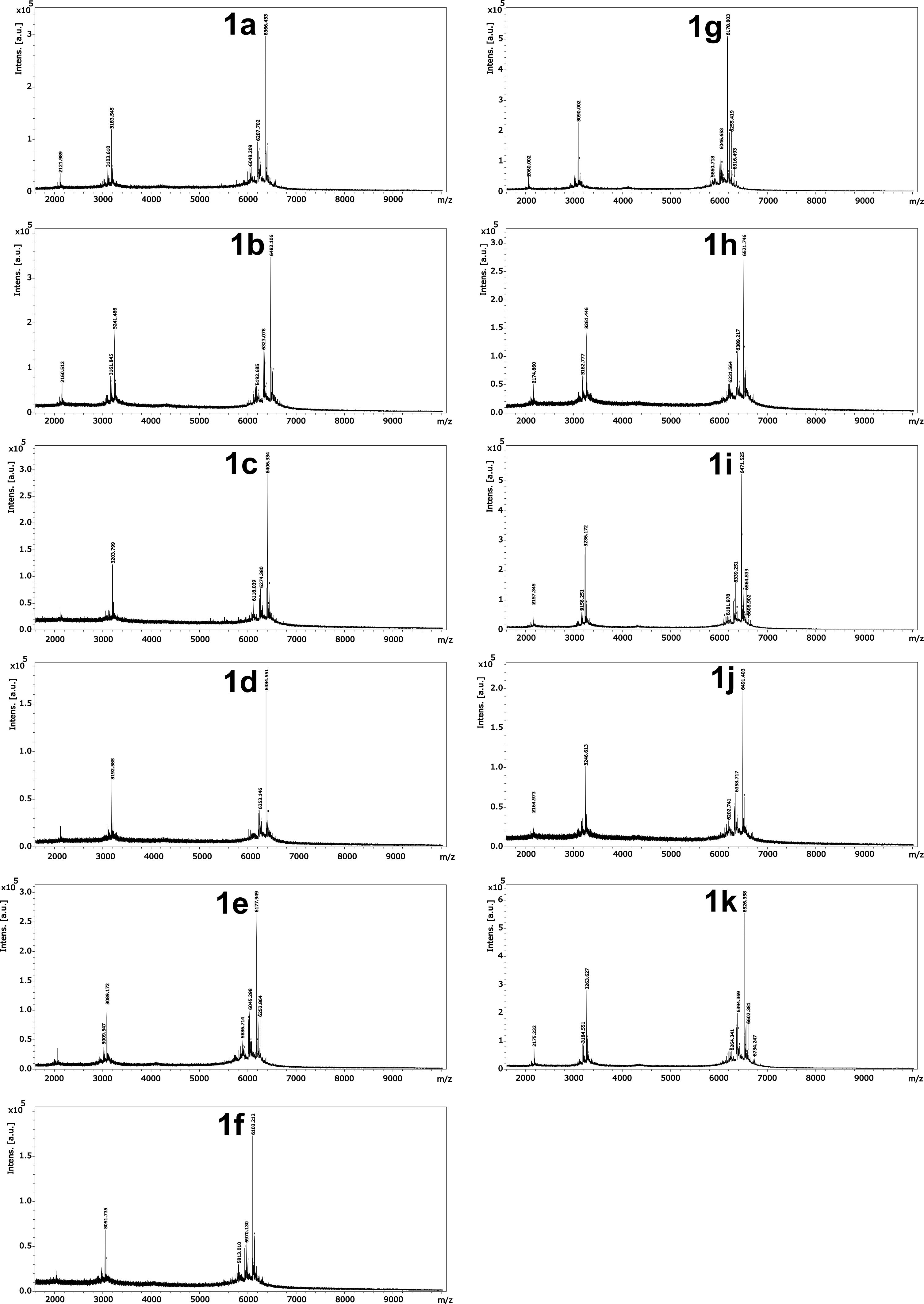

Supplement: S4 Fig — Indicated peaks represent [M + H] + of the expected m/z of each probe 1a-k. (TIF) [file pone.0318697.s004.tif]

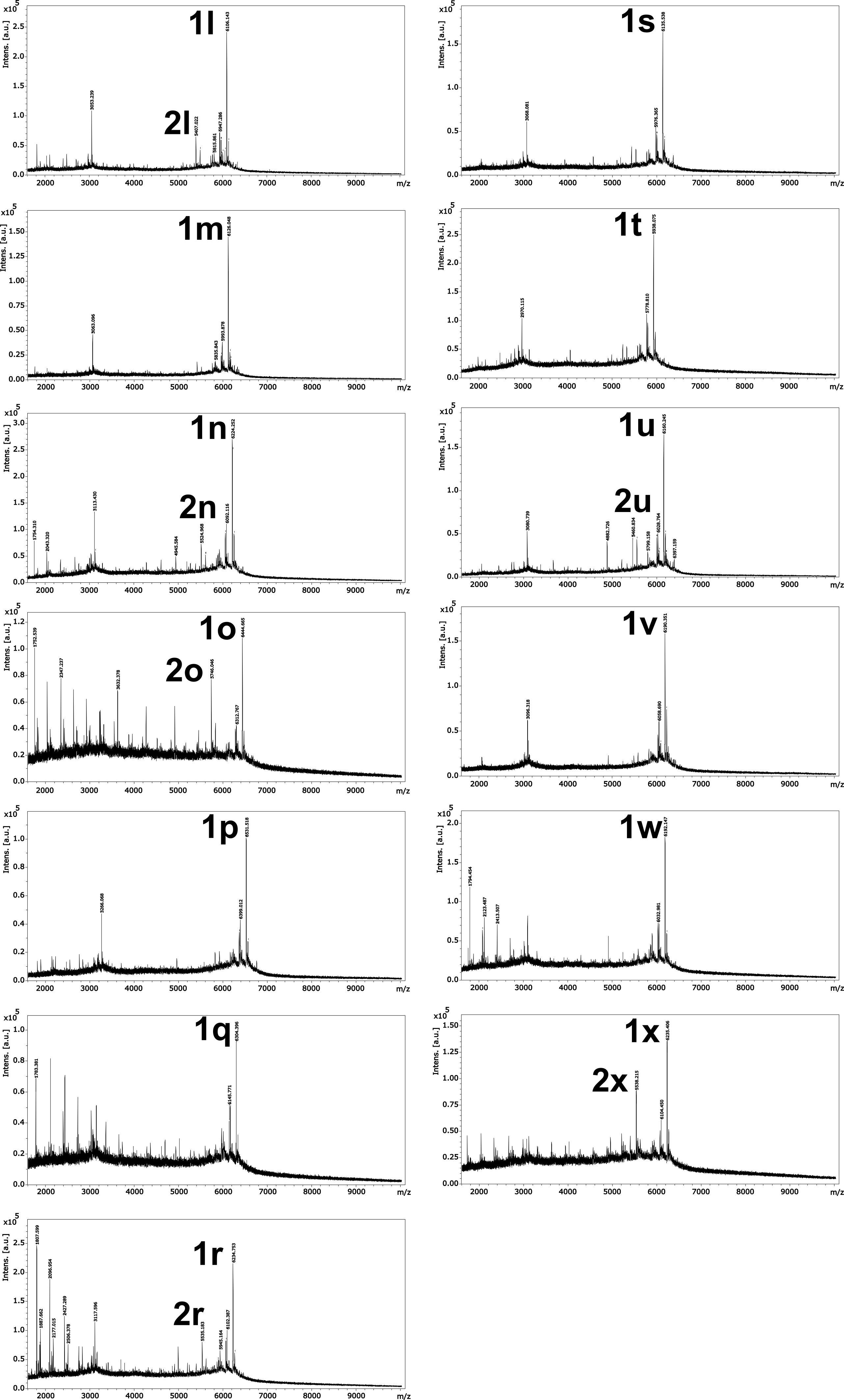

Supplement: S5 Fig — Indicated peaks represent [M + H] + of the expected m/z of each probe 1l-x.Not conjugated synthesis products were indicated as 2l-x. (TIF) [file pone.0318697.s005.tif]

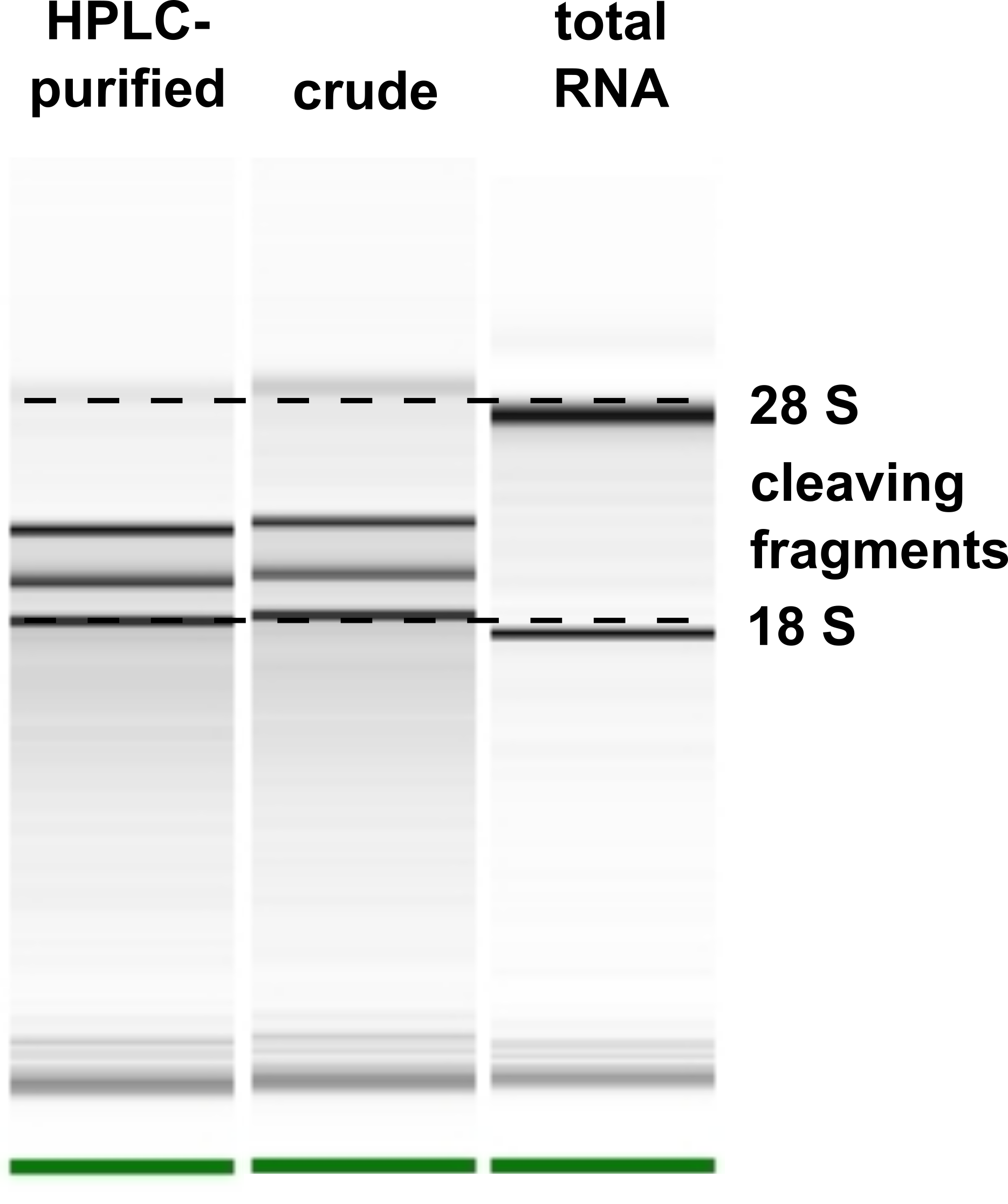

Supplement: S6 Fig — Total RNA was separated using a Agilent Bio Analyzer pico Assay. (TIF) [file pone.0318697.s006.tif]

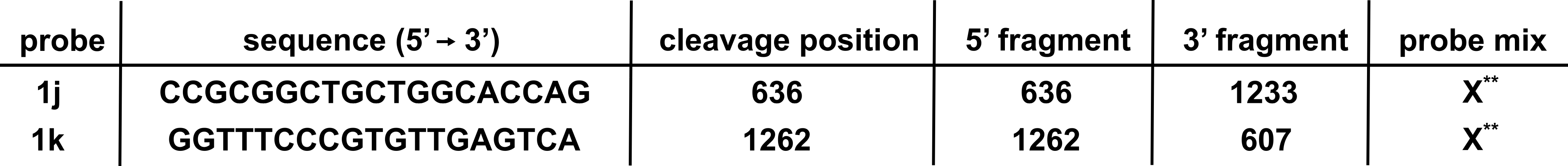

Supplement: S1 Table — 18S rRNA. ** indicates the absence in the not 18S targeting probe mixes. (TIF) [file pone.0318697.s007.tif]

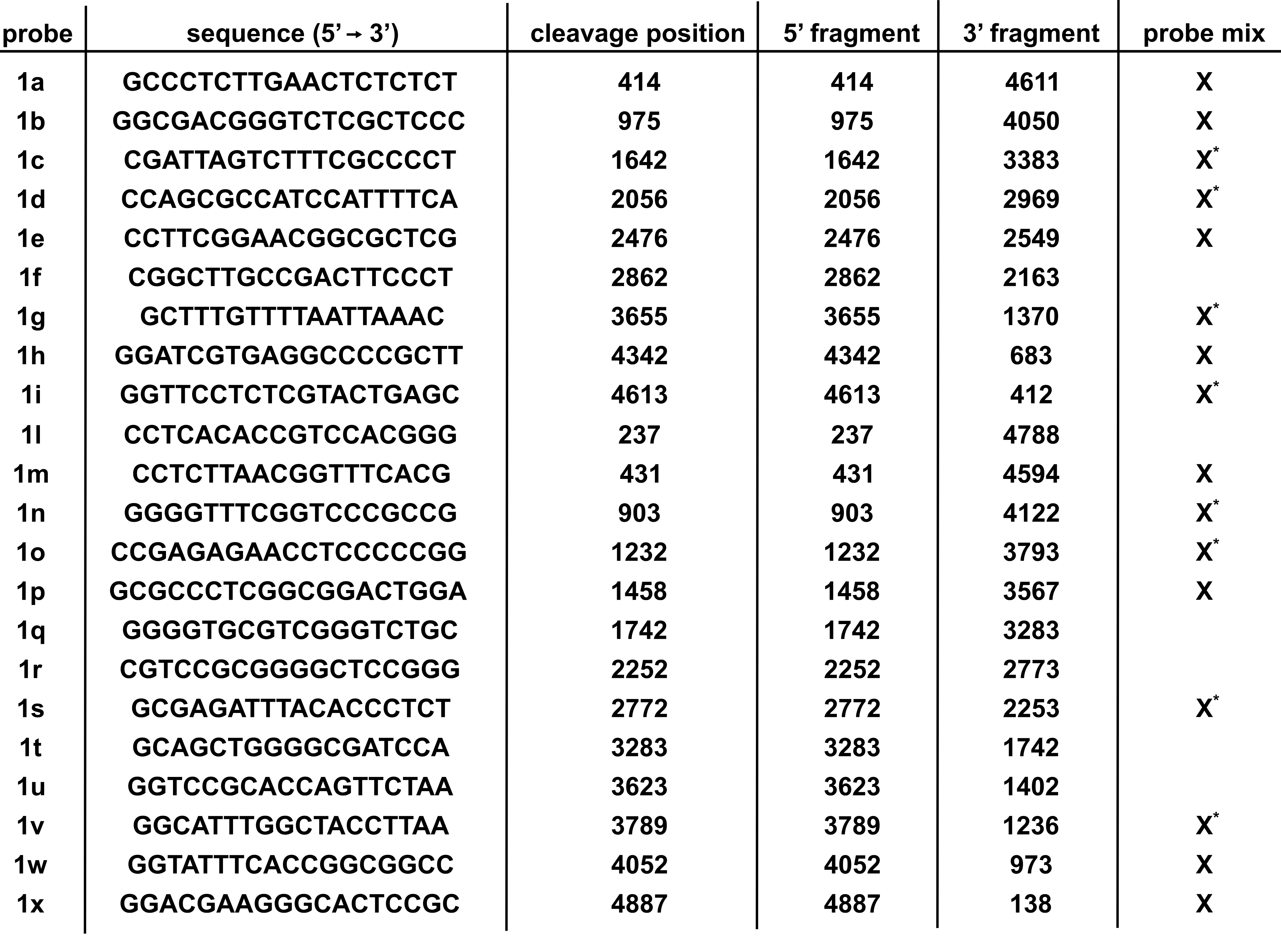

Supplement: S2 Table — 28 S rRNA. * indicates the absence of the probe in the 28 S probe mix v.2 (TIF) [file pone.0318697.s008.tif]
