# Supplementary material for: Chemical nucleases are a robust alternative for RNase H cleavage of human ribosomal RNA
Source: PLoS One. 2025 Feb 24;20(2):e0318697. doi: 10.1371/journal.pone.0318697 (PMC11849838; doi:10.1371/journal.pone.0318697)
Supplement: S1 raw images — (PDF) [file pone.0318697.s009.pdf]

Chemical nucleases are a robust alternative for RNase H cleavage of human ribosomal RNA

Hagen Wesseling[1,#], Dennis Krug[2,#], Marvin Wehrheim[1], Michael W. Göbel[2], Stefanie Kaiser[1,*]

Raw gel images

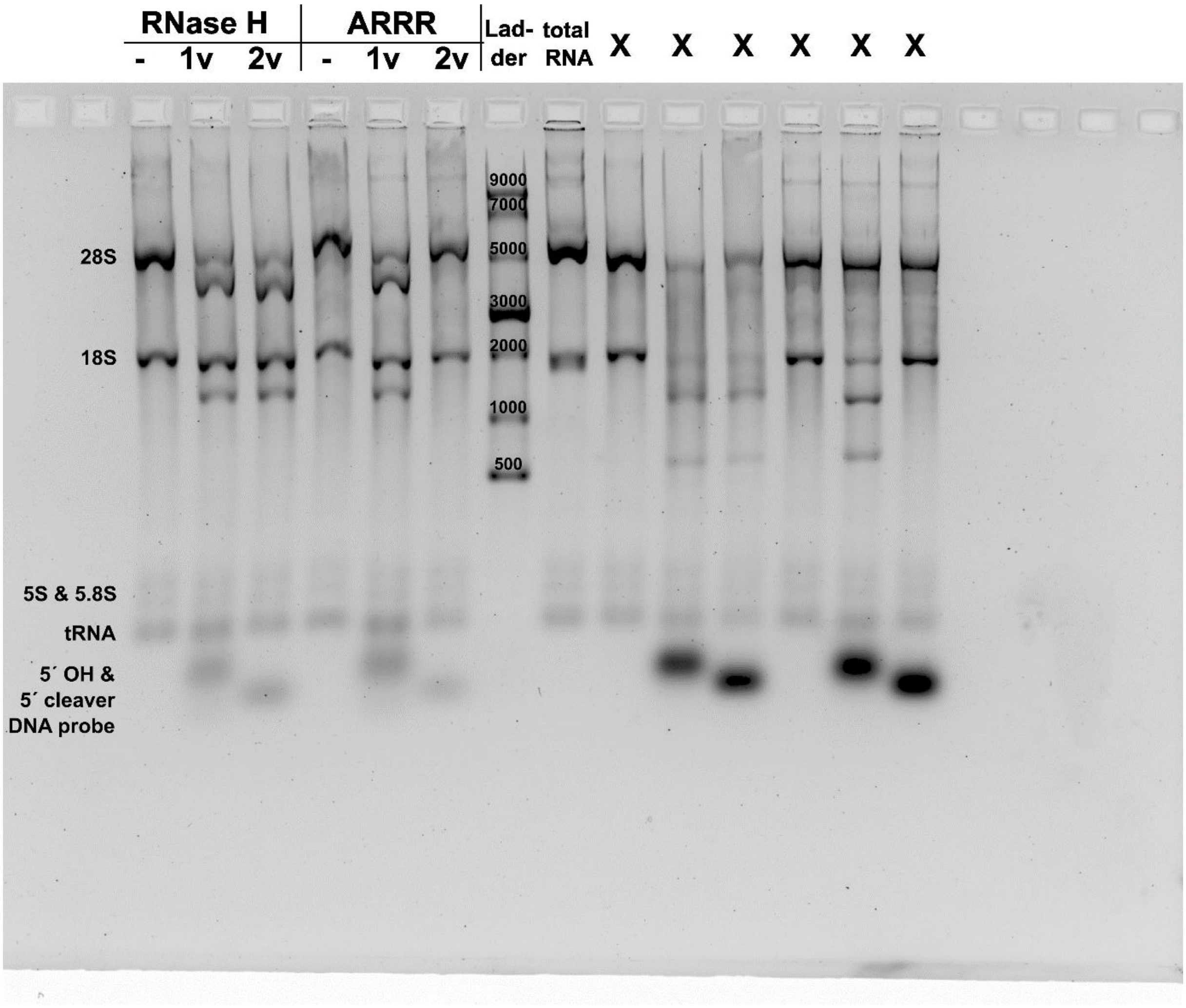

Original image Figure 2A: agarose gel image was cropped, brightened and mirrored for stronger data presentation

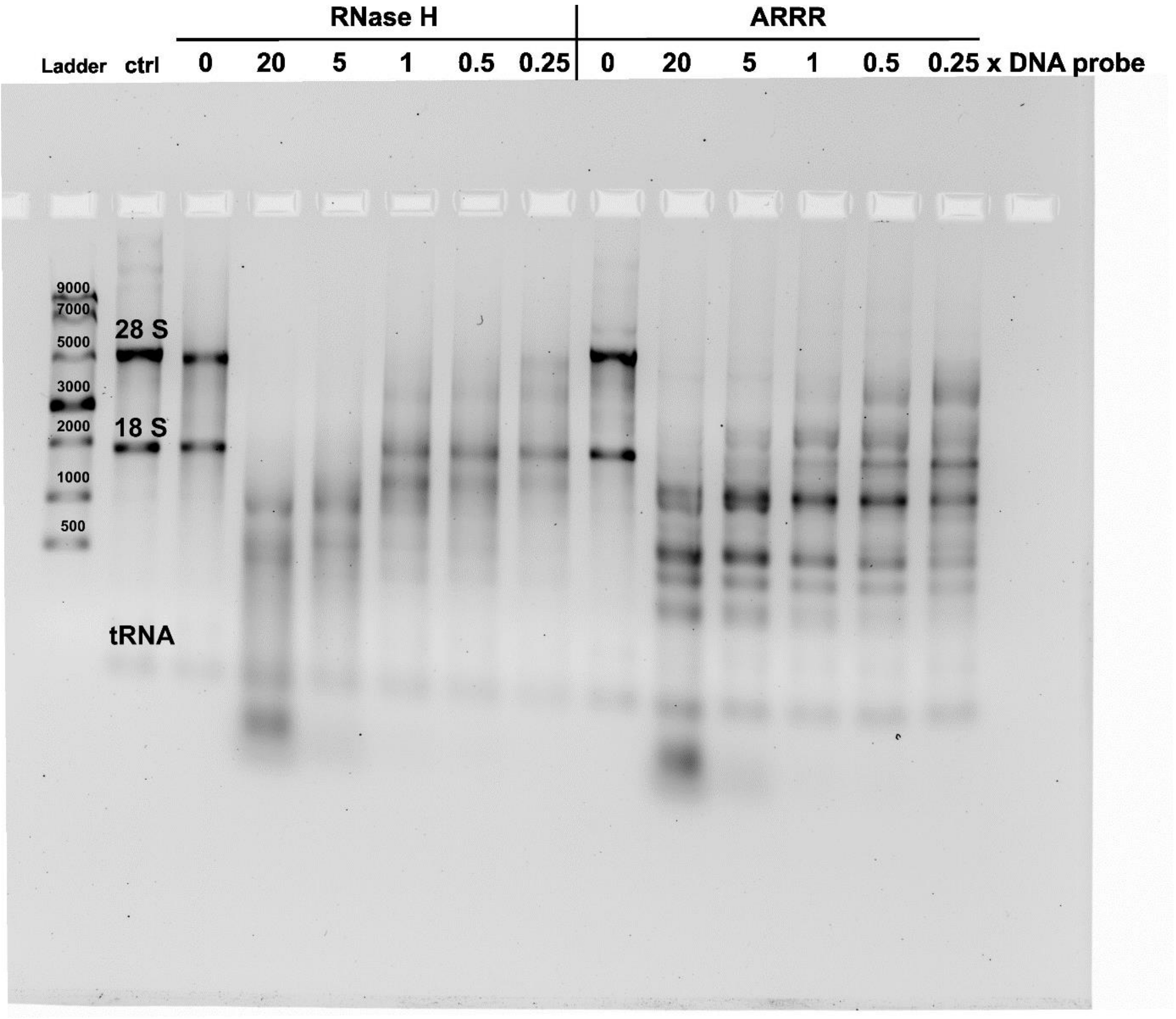

Original image Figure 3B: agarose gel image was brightened and cropped

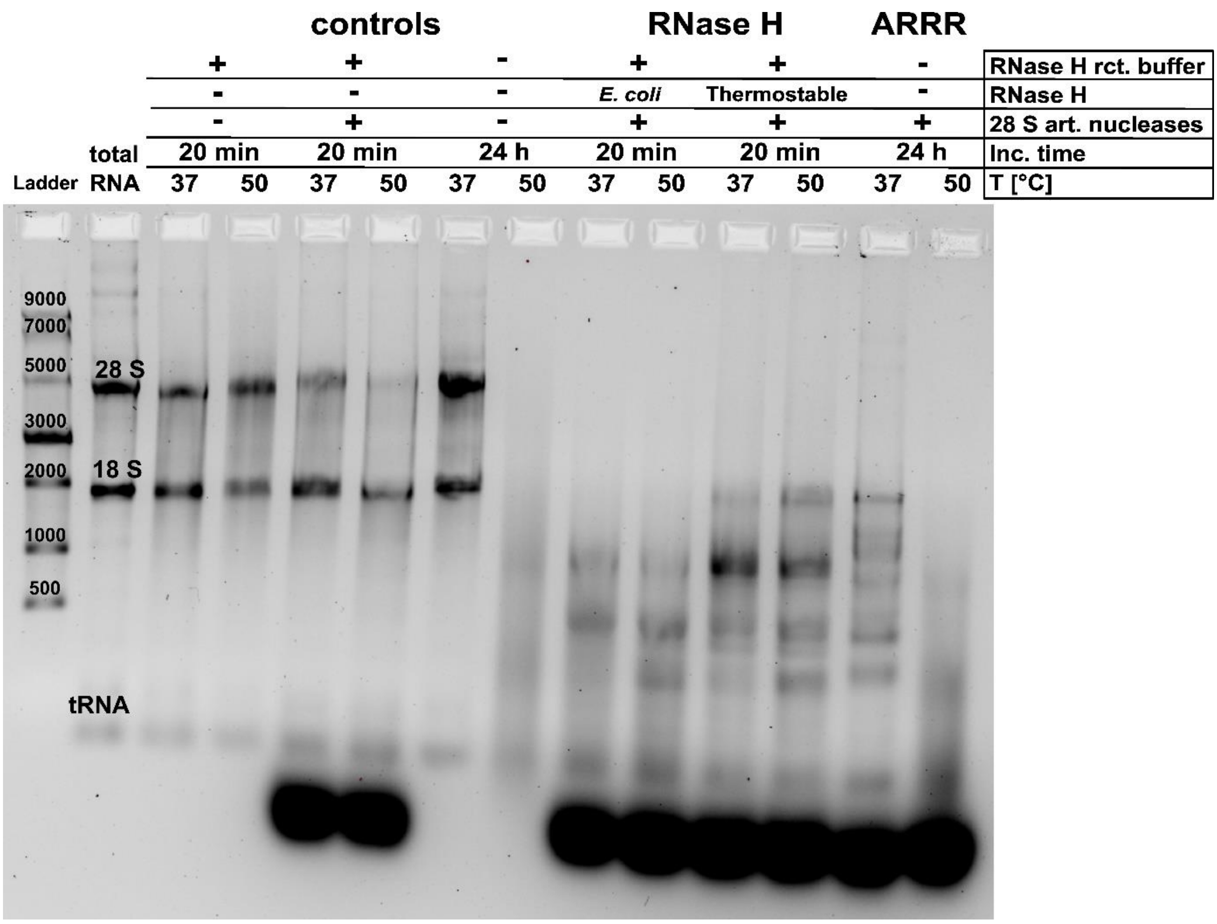

Original image Figure 3C: agarose gel image was brightened and cropped

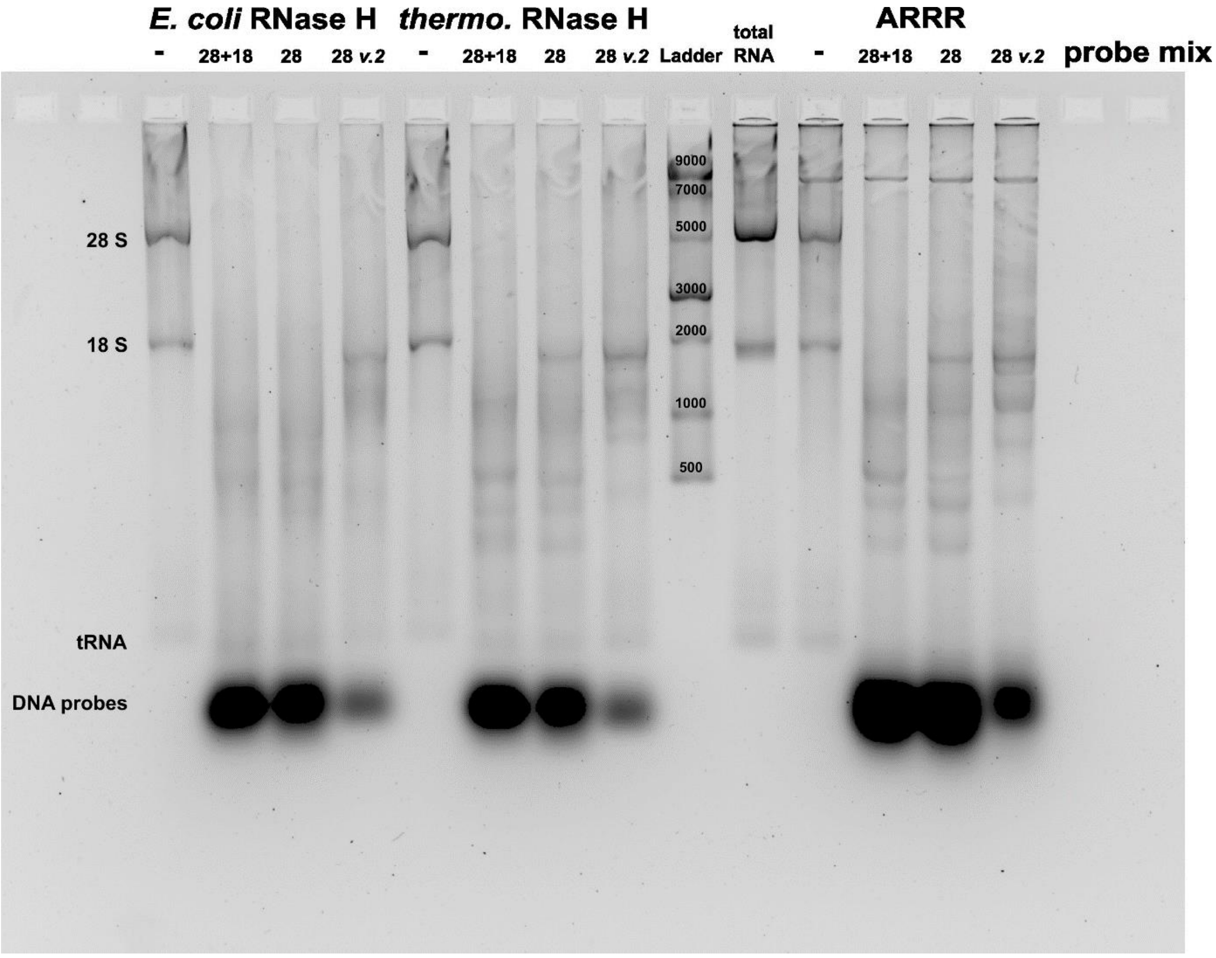

Original image Figure 4A: agarose gel image was brightened and cropped

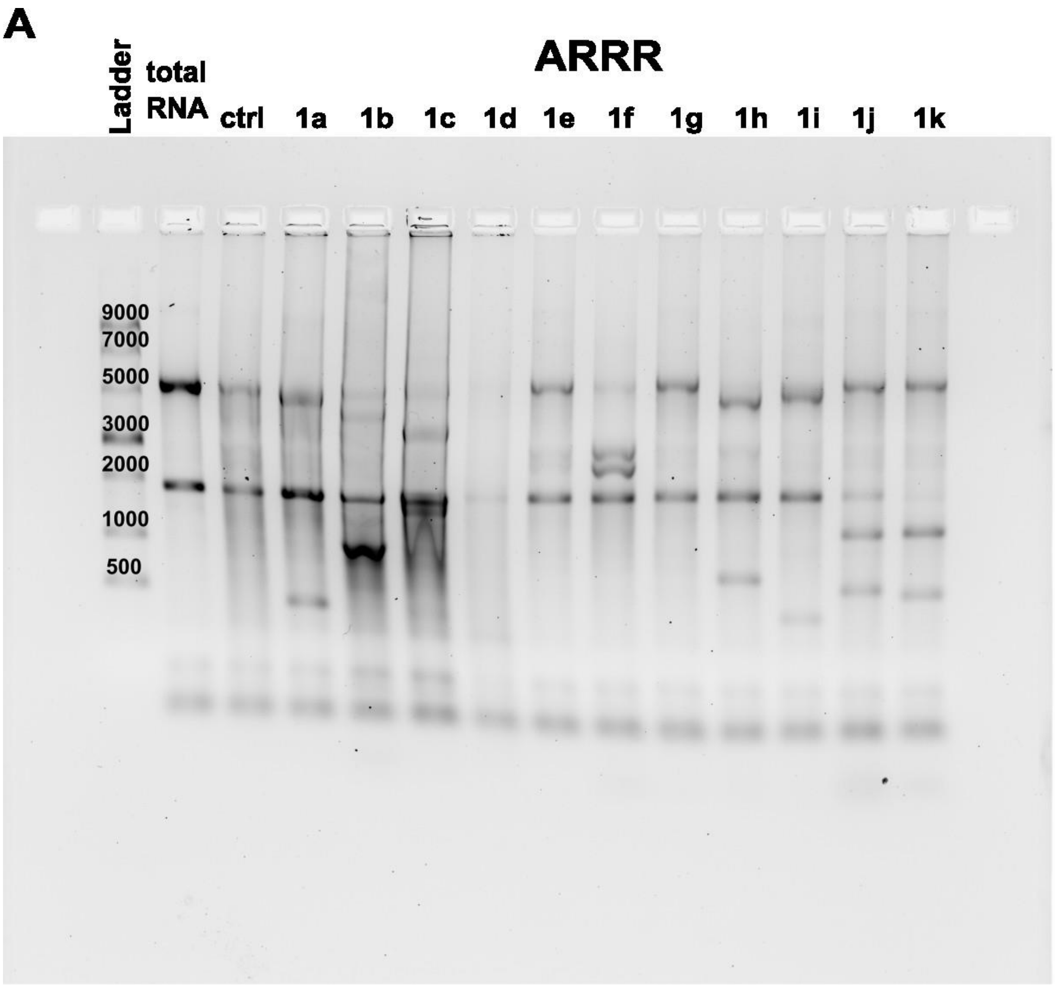

Original image S1A: agarose gel image was brightened and cropped

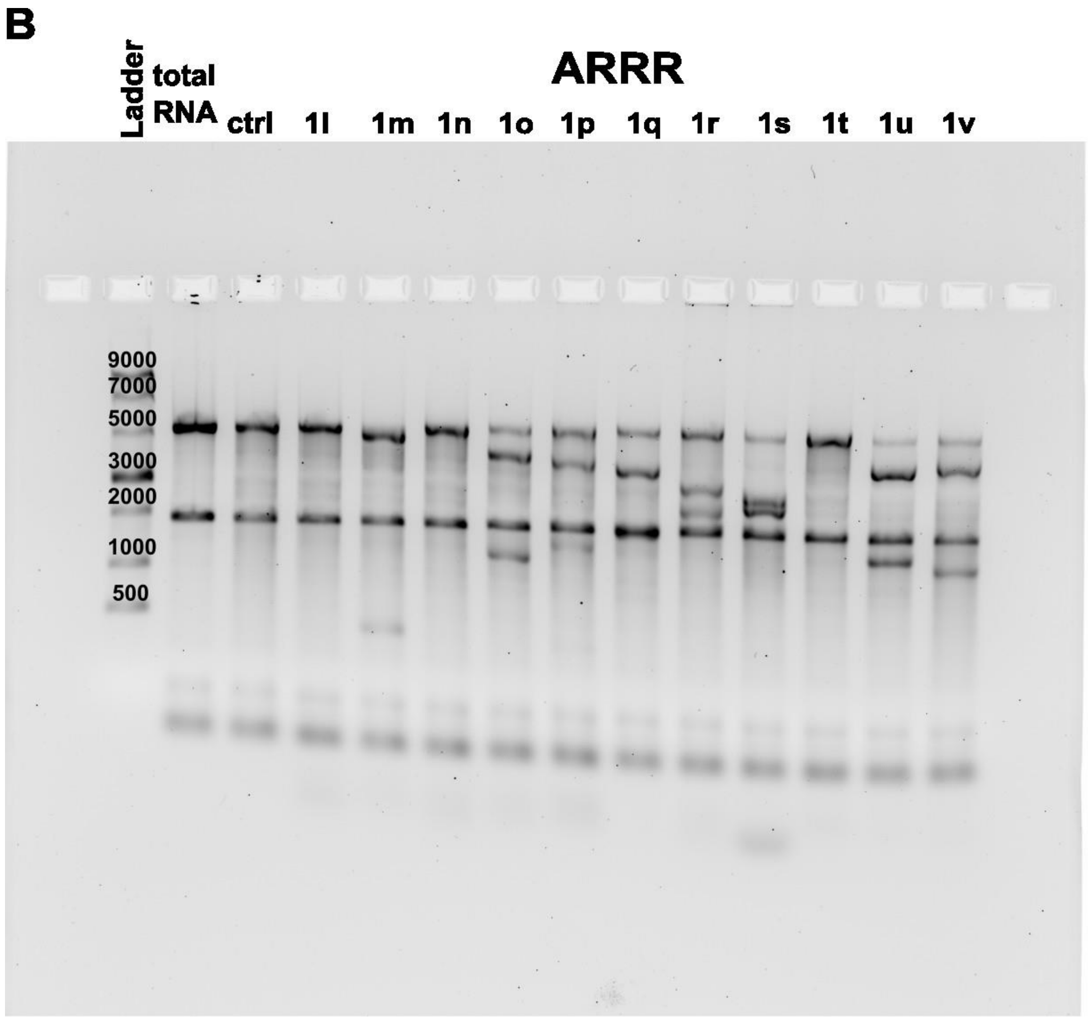

Original image S1B: agarose gel image was brightened and cropped

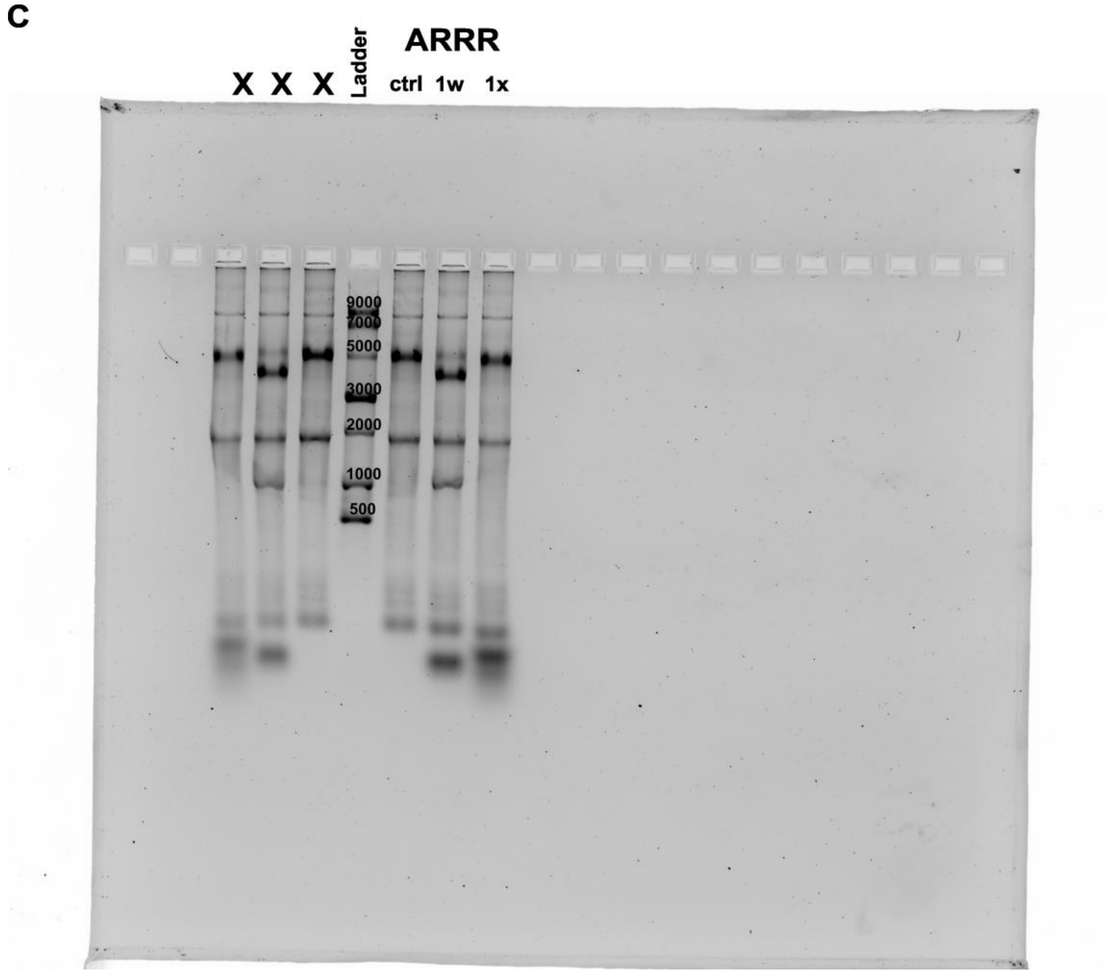

Original image S1C: agarose gel image was brightened and cropped

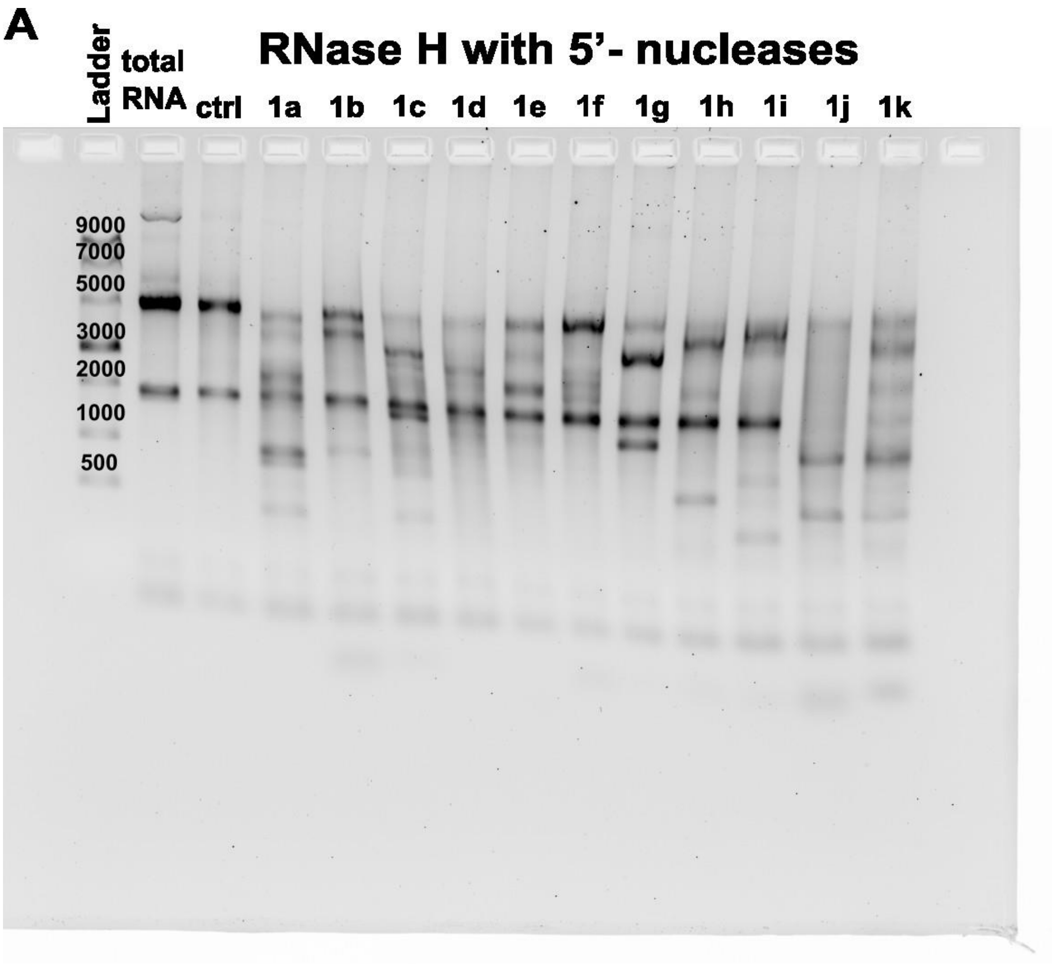

Original image Figure S2A: agarose gel image was brightened and cropped

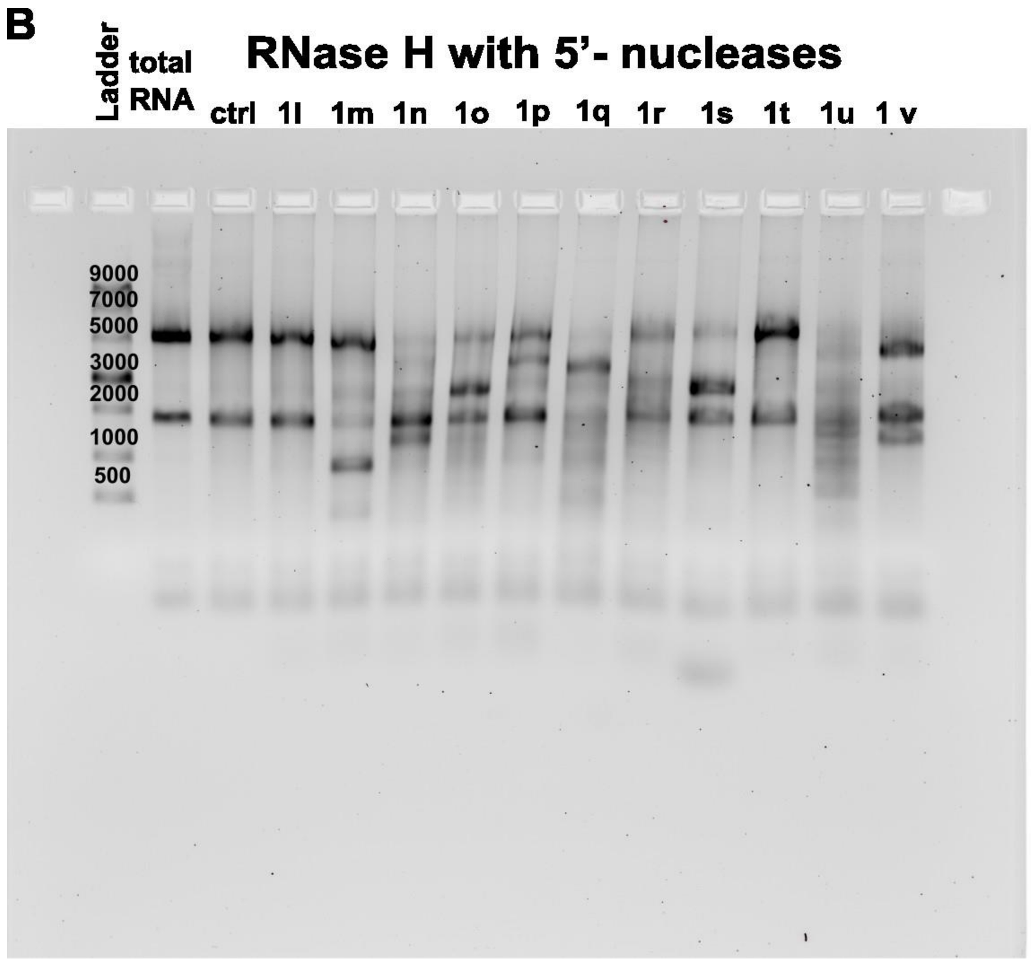

Original image Figure S2B: agarose gel image was brightened and cropped

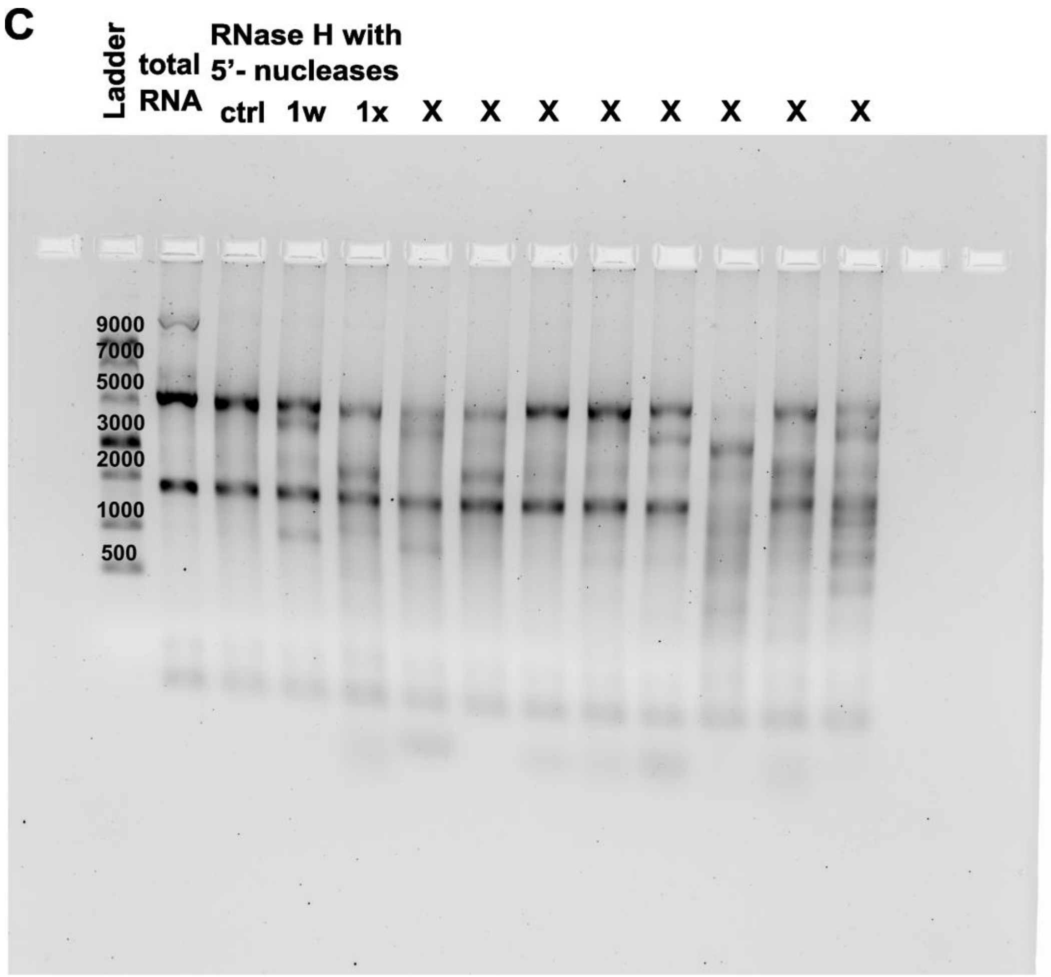

Original image Figure S2C: agarose gel image was brightened and cropped

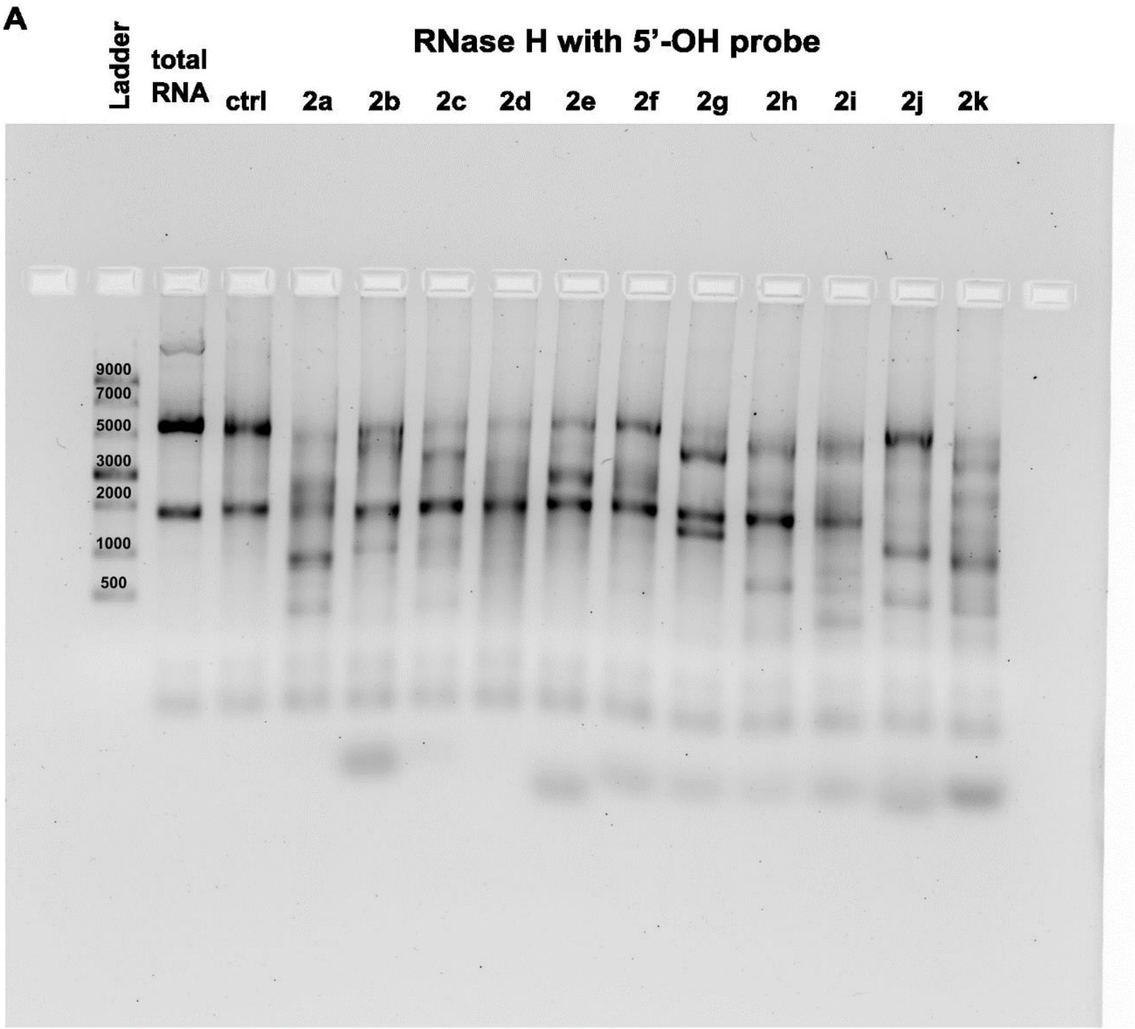

Original image S3A: agarose gel image was brightened and cropped

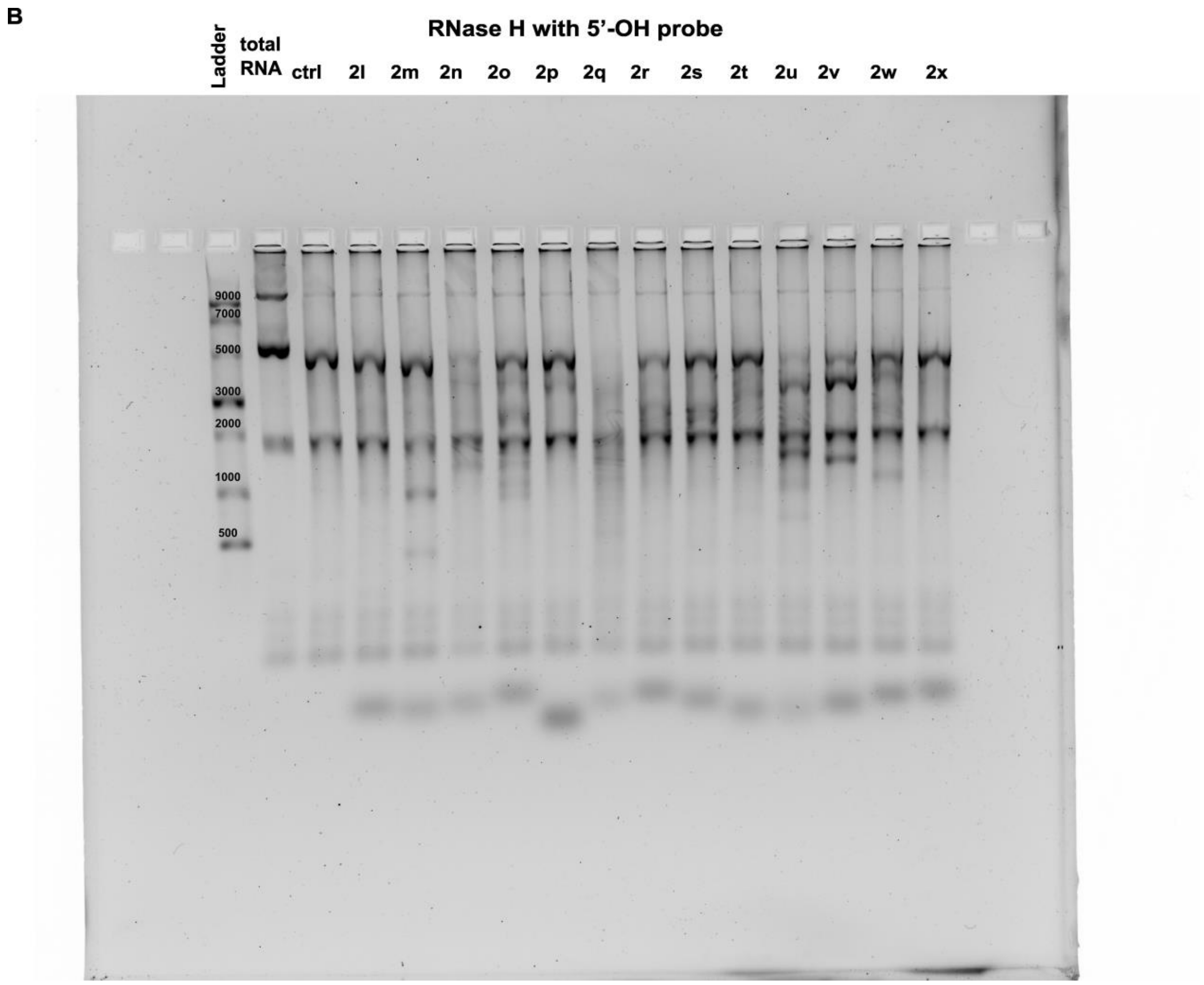

Original image Figure S3B: agarose gel image was brightened and cropped

Raw 2100 Bioanalyzer images

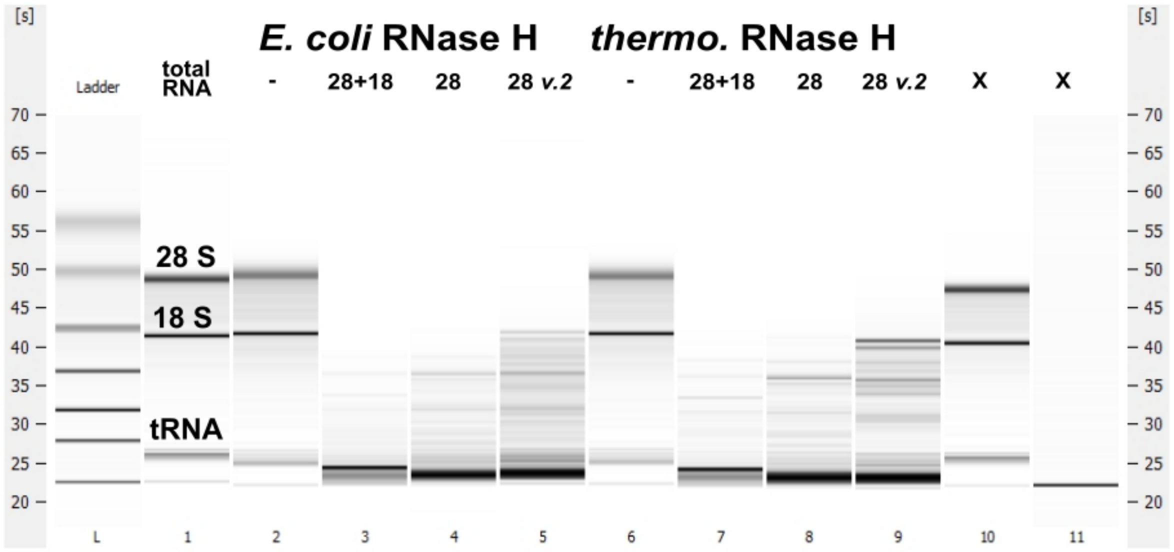

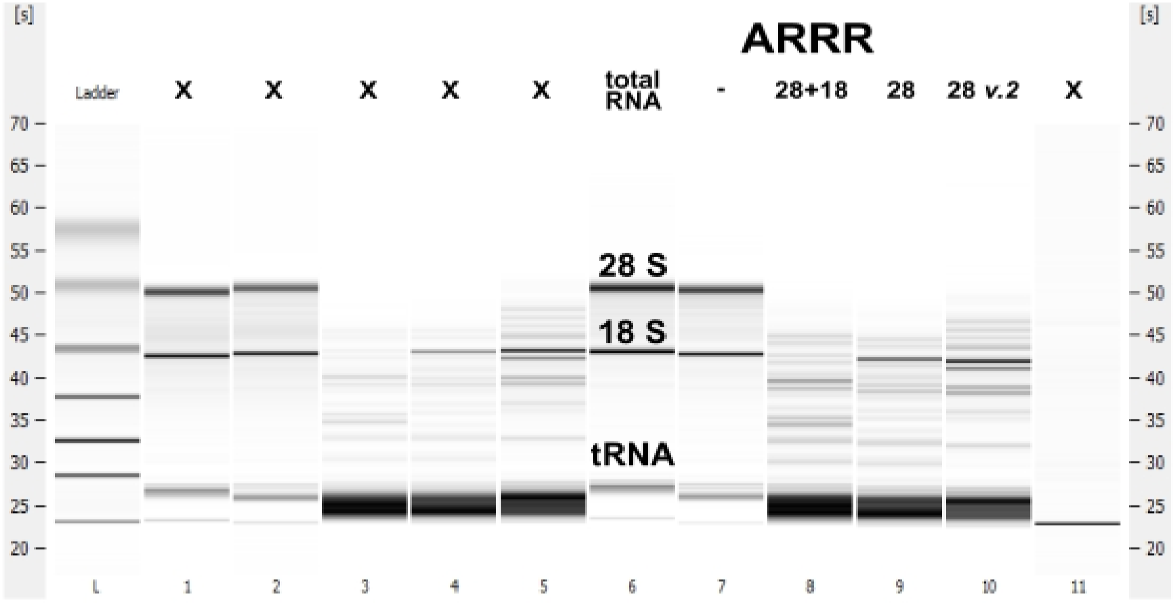

Original images Figure 4B: The figure at the top was cropped together with the figure at the bottom to display the experiments in parallel. The migration times of the controls (Ladder and total RNA) are consistent across both Bioanalyzer assays.

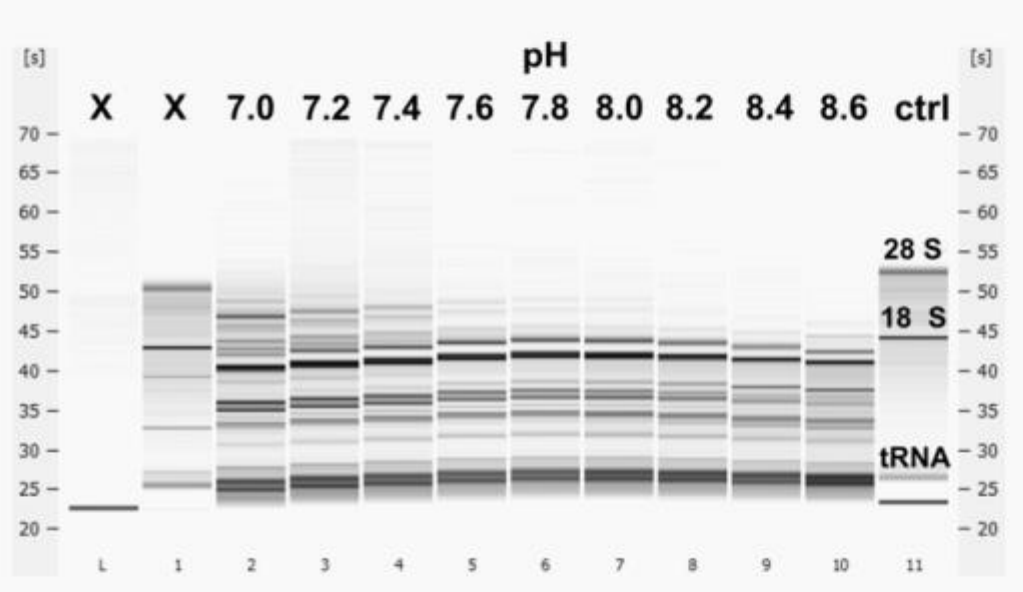

Original Image Figure 3A: Figure was cropped

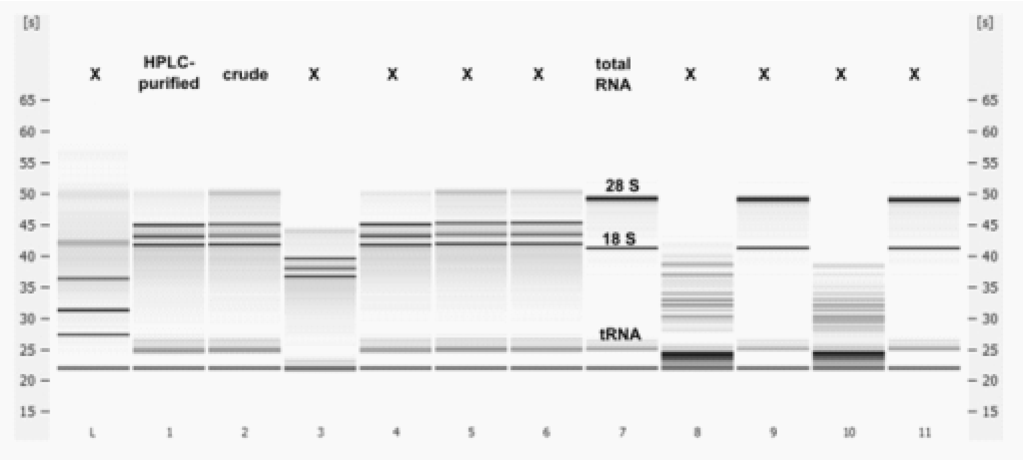

Original Image S6 Figure : Figure was cropped
