# Supplementary material for: Chemical nucleases are a robust alternative for RNase H cleavage of human ribosomal RNA
Source: PLoS One. 2025 Feb 24;20(2):e0318697. doi: 10.1371/journal.pone.0318697 (PMC11849838; doi:10.1371/journal.pone.0318697)
Supplement: Attachment — Submitted filename: point by point response Wesseling.docx [file pone.0318697.s011.docx]

We thank the reviewers for their time and expert opinion on our manuscript. Our detailed point-by-point-response is indicated by * and blue font.

**Reviewer #1**

In this work, Wesseling et al. present the early stages of proof-of-concept work for the use of RNA-cleaving DNA probes in the depletion of rRNA for RNA-sequencinng and mass-spec applications. The idea has some merit, as shown by the limited studies presented here. The authors have not yet demonstrated its suitability for these tasks, but this is not a prerequisite for publication.

* We thank the reviewer for the assessment of our manuscript and the comments that have improved the quality. We will give a detailed point-by-point response below.

I think the manuscript could be strengthened if the following questions/comments were considered:

1) Perhaps the title and abstract could be changed to better reflect the non–protein nature of the “artificial nucleases”. I did not realize that these nucleases were DNA probes with cleavage-inducing chemical groups until the end of the introduction. I was expecting some sort of engineered enzyme until I reached that point.

* We thank the reviewer for this suggestion. We added in the abstract that the artificial nucleases consist of DNA probes linked to a small-molecule chemical nuclease and instead of “artificial” we now call them “chemical” nuclease. Thus, the title has changed.

2) I am concerned by the use of native TAE gels for these analyses. The rRNA is extremely structured in nondenaturing buffers, even after being previously denatured, and this structure can hold the rRNA together even after cleavages have occurred. A denaturing gel or other denaturing method should ideally be used. Similarly, I wonder if the fuzzier bands in the RNase H treatment in 3B could be due to RNAseH remaining bound to the RNA and affecting its mobility.

* We sincerely thank the reviewer for their thoughtful consideration of our experiments. While we are confident that heat denaturation should be sufficient to disrupt the secondary structures of rRNA, we also considered this potential bias early on in this project. Therefore, many preliminary data (not included in the initial submission), were done with chip gel electrophoresis (Agilent Bioanalyzer) that utilizes a denaturing gel matrix/buffer system. Here, we soon learned that results from the denaturing, but expensive chip electrophoresis are identical to results obtained by cost-efficient agarose electrophoresis. To address the reviewer’s concerns, we now include a BioAnalyzer gel of the final comparison in Figure 4B (see next page for your convenience).

**Fig 4: Targeted cleavage of 28 S rRNA reveals that ARRR has little off-target effect**. **A** Total RNA was treated with an all rRNAs cleaving probe mix (28+18), a 28 S specific probe mix (28) or the 28 S specific mix where potential 18 S targeting probes were excluded (28 v.2). RNAs were separated on an 2% Agarose-TAE gel and stained with GelRed. **B** The same experiment as in A was loaded on an Agilent BioAnalyzer pico chip for denaturing chip gel electrophoresis.

3) Several commercial RNaseH based kits for RNA–seq exist at relatively low cost (from Illumina and NEB for example), as well as low–cost DIY methods (See Culviner et al, mBIO 2020). These methods are clearly suitable for basic RNA–seq, so the authors’ claim of high prices and that specialized modified oligos are required does not seem to be correct. However there may be more specialized cases where the off-target activity of RNaseH is a bigger issue (for example see Zinshteyn et al. RNA 2020).

* We thank the reviewer for this comment that indicates that we did not clearly communicate the purpose of our project. As a mass spectrometry-focused lab, we often encounter limitations with commercial kits due to insufficient input amounts. For example, the two mentioned RNase H kits are designed for a maximum input of 1 µg total RNA (or poly-A enriched RNA) which yields single-digit nanogram amounts of mRNA. While this might be sufficient for sequencing, it is not for quantification of RNA modifications even for abundant modifications such as m^6^A. For this reason, our lab uses a combination of biotin-streptavidin- pulldown assays, first to enrich poly(A) RNA and subsequently to remove rRNA (Hagelskamp et al, RSC Chemical Biology 2023, Ito-Kureha et al, Nature Immunology, 2022, etc.). With this method a near complete removal of rRNA is possible. The cost per sample for using both of the commercially available kits is approximately 250 €. The main challenge we and other mass spectrometrists face is that a single round of rRNA pulldown - whether in our hands or as reported by Culviner et al. (mBIO 2020) - results in approximately 20% residual rRNA contamination. This level of contamination is unacceptable for nucleoside mass spectrometry. These challenges prompted us to explore chemical nucleases and compare their performance with RNase H, ultimately leading to the discovery of the off-target effects associated with RNase H.

We appreciate the comment of the reviewer and we have changed the manuscript text to emphasize our motivation. Ultimately, if chemical nucleases are equally or better suited to degrade rRNA, the prize of rRNA depletion will drop which will benefit not only MS work but also sequencing.

4) In figure 4, what is the high molecular weight band above the 28S only in the ARRR lanes? Why does the supposed 18S band shift lower after ARRR treatment? Are the authors sure that this is not a 28S degradation product or a partially cleaved 18S?

* We thank the reviewer for their careful examination of our figures. Unfortunately, we do not have a definitive explanation for the band observed above the 28S rRNA in the ARRR lanes on the agarose gel. All experiments were conducted in parallel using the same total RNA as the starting material. The band is present even in the absence of the chemical nucleases. Here, the total RNA is mixed with the reaction buffers and thus we conclude that the buffer components are involved of the band formation. We notice that the band is not affected by the chemical nucleases which excludes pre-rRNA as the origin. Although we have no explanation of this band at the current stage, we decided to show the full gel and not crop it. We hope the reviewer can agree on our decision and accepts that the band is potentially not important for the functional interpretation of the chemical nucleases.

Regarding the second point: Given that the intensity of the supposed 18S band increases as we protect the 18S rRNA more effectively, we are confident that this band indeed represents the 18S rRNA. We observe similarly altered migration patterns in the controls analysed using the Bioanalyzer, and we hypothesize that these shifts may result from the addition of the high ionic strength buffers used in the respective assays.

5) Is there a roadmap for further optimizing the reactivity and specificity of these compounds? The requirement for 24-hour incubations in ph8 buffer seems like a risky and time-consuming requirement, and a serious impediment too widespread use

* In the non-conjugated form, when compared to 2-aminobenzimidazoles, 2-aminoimidazoles are considerably faster RNA cleavers (F. Danneberg, H. Westemeier, P. Horx, F. Zellmann, K. Dörr, E. Kalden, M. Zeiger, A. Akpinar, R. Berger, M. W. Göbel, Eur. J. Org. Chem. 2021, 6358–6366). Unfortunately, we have not yet completed the synthesis of the related oligonucleotide conjugates, but we consider expanding the chemical toolbox to advance the chemical nucleases for the application. Furthermore, we are currently developing multiple-turnover conjugates which will also speed up the process.

6) At several points in the conclusion, the word “prize” is used instead of price.

* We thank the reviewer for his input and correct the word in the manuscript.

**Reviewer #2**

The authors proposed a new method for cleavage of human ribosomal RNA. The proposed method is promising and applicable. However, I have a few concerns.

* We thank the reviewer for the assessment of our manuscript and the comments that have improved the quality. We will give a detailed point-by-point response below.

Major concerns
1. This paper does not discuss the stability or reproducibility of their proposed system. I suggest adding some replicates and test the stability of their method.

* We thank the reviewer for their valuable suggestion. We decided to include only the gels that ran perfectly in the manuscript. (Perfectly in terms of perfect handling without parts breaking off, even staining, equal migration patterns etc.) In preliminary experiments, we tested different run times and agarose concentrations and ultimately chose to present only those that adhered to the same experimental conditions (2% agarose, 2-hour run time). Previous gels from less-concentrated gels and varied run times exist, show the same experimental result, but for consistency were not included in the manuscript. We hope to address the reviewer’s concerns by including a newly added Bioanalyzer chip with fresh samples, which we believe further demonstrates the reproducibility of the key experiment. Given that we already present 16 gels (including Bioanalyzer chips), we feel that adding more gel replicates would overly complicate the Supporting Information. However, if the reviewer would still like to see additional gels, we have several that were excluded from the manuscript due to minor imperfections and would be happy to share them.

2. The authors argued that one major drawback of the RNase H-based kits is its high price. How about the price of the newly proposed method?

* We thank the reviewer for this comment. Regarding the price of the chemical nucleases, the amidite building blocks are not commercially available, so we currently synthesize the chemical nucleases on a small scale within the scope of academic research. It is difficult to predict how pricing would evolve if large-scale production were to be adopted by commercial suppliers. But as we now developed chemistries that run on standard DNA synthesizers, we expect the price to match other common modified bases e.g. biotin-tags etc.

Minor concerns
1. In the abstract, the authors claimed that they "find no unspecific cleavage of non-target RNA". But in the Result section, they only concluded "less unspecific cleavage" by their method (page 6). I think the claim in the abstract is too strong.

* We thank the reviewer for point out this unintended inconsistency. We corrected the abstract.
